# The role of OsDGD1 in phosphate starvation: How lipid remodeling regulates jasmonic acid and root development in rice

**DOI:** 10.1093/plphys/kiae524

**Published:** 2024-10-04

**Authors:** Alaeddine Safi

**Affiliations:** Assistant Features Editor, Plant Physiology, American Society of Plant Biologists; Department of Plant Biotechnology and Bioinformatics, Ghent University, B-9052 Ghent, Belgium; VIB Center for Plant Systems Biology, B-9052 Ghent, Belgium

Phosphorus is an indispensable macronutrient for sustaining life as it is a key component of cell membranes and nucleic acids and is essential for energy metabolism and signaling processes. Plants acquire and utilize phosphorus mainly in the form of phosphate ions known as inorganic phosphate (Pi). However, the physicochemical properties of Pi make it poorly available for plant acquisition. Its pronounced tendency to form complexes with cations (e.g. Al, Ca, Fe) and organic molecules (e.g. in the form of inositol phosphates) reduces its solubility and mobility in the soil (Lopez-Arredondo et al. 2014).

To overcome the scarcity and fluctuations in Pi bioavailability, plants rely on the Pi starvation response (PSR), consisting of a range of adaptive physiological and developmental processes that enhance Pi uptake and phosphorus use efficiency (PUE) (Medici et al. 2019).

At the developmental level, root growth is prioritized over the shoot during PSR, and the plant invests in root branching in a quest for Pi, which is more abundant in the topsoil. Having more and longer lateral roots allows the plant to explore a larger space in the surrounding environment. Lateral roots are covered by dense and long root hairs, increasing the root surface area in contact with the rhizosphere and thus the Pi absorption capacity (Lopez-Arredondo et al. 2014).

The physiological aspects of PSR include reduced photosynthesis rate and the induction of Pi starvation-induced (PSI) genes such as Pi transporters to increase Pi uptake and translocation. PSI genes are tightly regulated by the GARP transcription factors known as Phosphate Starvation Response (PHR) proteins (Safi et al. 2017). During PSR, plants also produce root exudates containing enzymes (e.g. phosphatases) and organic acids (e.g. malate, citrate) that help release Pi from organic compounds and metallic clusters, respectively. Internally, plants enable source-to-sink phosphate remobilization toward actively growing tissues. At the cellular level, phosphorus recycling is enhanced through autophagy and membrane lipid remodeling. The latter involves the substitution of membrane phospholipids, which contain up to 30% of the cell organic phosphorus content, by phosphorus-free lipids such as galactolipids and sulfolipids. Mutants with lower sulfo- or galactolipid contents have growth defects in Pi-deficient conditions (Verma et al. 2022).

Monogalactosyl diacylglycerol (MGDG) and digalactosyl diacylglycerol (DGDG) are the predominant galactolipids in plants (Dormann and Benning 2002). They are respectively synthesized by MGDG and DGDG synthases (namely MGD and DGD) (Fig. 1). Previous studies on Arabidopsis and rice revealed the importance of a number of *MGD* and *DGD* genes encoding isoforms of these enzymes in plant growth and resilience towards Pi deprivation (Verma et al. 2022). Interestingly, MGDG and DGDG not only serve as building blocks of membranes but also as a source for fatty acid precursors used in jasmonic acid (JA) synthesis. Moreover, the MGDG to DGDG ratio influences JA accumulation and signaling (Lin et al. 2016; Yu et al. 2020).

**Figure 1.**
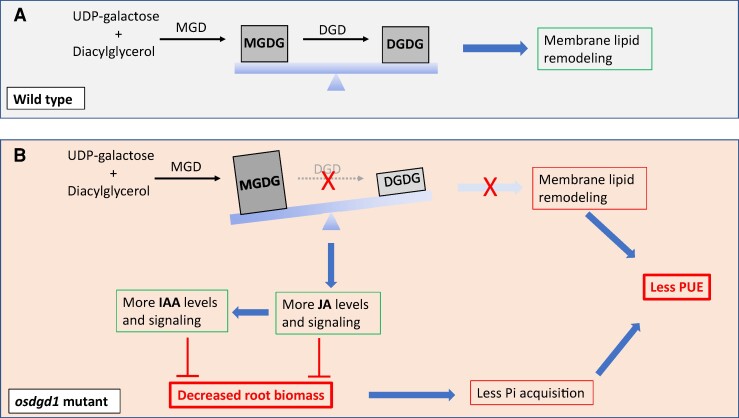
Proposed model highlighting the importance of maintaining a balanced MGDG to DGDG ratio in plant nutrition and growth, especially under Pi starvation conditions. **A)** The normal response to Pi deprivation in wild-type plants. This response includes an induction of MGDG and DGDG synthesis leading to membrane lipid remodeling. **B)** However, when the *DGD* gene is mutated (*osdgd1*), the MGDG to DGDG ratio is disturbed. The imbalance in the MGDG to DGDG ratio not only affects membrane remodeling, which leads to less PUE, but also affects the hormonal homoeostasis. The higher MGDG to DGDG ratio enhances JA biosynthesis, which in turn induces auxin accumulation. The increased levels and signaling of both hormones repress the primary root growth. The shorter roots and decreased root biomass negatively affect the mutant Pi uptake and therefore its PUE.

In a recent study published in *Plant Physiology*, Pandey et al. (2024) explored another gene involved in this metabolic pathway: *OsDGD1*. *OsDGD1* is 1 of 5 rice *DGD* genes that catalyze the conversion of MGDG to DGDG. It was found to be highly and specifically induced by Pi deficiency but not by other nutrient limitation (Pandey et al. 2024).

To characterize *OsDGD1* function, the authors generated 2 independent knockout mutants using CRISPR/Cas9. *osdgd1* lines have an impaired root and shoot growth biomass associated with weakened Pi acquisition and utilization. These growth defects are more pronounced during Pi deprivation. An extended examination of the mutant roots revealed a shorter primary root as a result of a smaller meristem, which is where *OsDGD1* is predominantly expressed (Pandey et al. 2024). Additional investigations showed changes in lipid composition in the *osdgd1* mutants, especially a decrease in DGDG and an increase in MGDG content (Pandey et al. 2024).

Because the MGDG to DGDG ratio affects JA accumulation and signaling (Lin et al. 2016), the imbalance in MGDG and DGDG contents in *osdgd1* mutants prompted the authors to explore JA status. They noticed a rise in the expression JA biosynthesis and signaling genes (e.g. *OsLOX2*, *OsMYC2*). As expected, the upregulation of JA biosynthesis genes contributed to the accumulation of higher levels of JA and its conjugated bioactive form JA-isoleucine, which might be the reason behind the mutant short root phenotype (Fig. 1B). Indeed, treatment of *osdgd1* with sodium diethyldithiocarbamate (a JA biosynthesis inhibitor) restored the phenotype of the primary root and root meristem. Furthermore, exogenous application of increasing concentrations of methyl jasmonate (MeJA) correlated with higher inhibition of root growth in wild-type plants. The *osdgd1* mutants displayed a much milder response to MeJA supply due to an already high endogenous JA content (Pandey et al. 2024).

Given the central role of auxin in root development (Chen et al. 2021) and the crosstalk between JA and auxin in the context of Pi deprivation (Zheng et al. 2021), Pandey and colleagues assessed the dynamics of auxin-related genes. In wild-type plants upon MeJA treatment, the authors observed an increase in the expression of auxin biosynthesis, transport, and signaling genes (e.g. *OsTAR1*, *OsYUC8*, *OsPin1b*, *OsIAA20*) as well as in DR5 promoter activity. Interestingly, the same trend was seen when comparing untreated *osdgd1* and wild-type plants, with an even higher number of auxin-related genes being upregulated in the mutants. Therefore, the augmentation in auxin (IAA) content and its related genes in the *osdgd1* knockout lines is most likely due to the endogenously elevated levels of JA (Pandey et al. 2024) (Fig. 1B).

In conclusion, this study sheds light on the role of a rice lipid synthesis enzyme in maintaining phosphorus homeostasis and plant growth. Pi starvation triggers membrane lipid remodeling and the replacement of phospholipids with Pi-free alternatives such as galactolipids. OsDGD1 is an important enzyme involved in galactolipid synthesis by transforming MGDG into DGDG. Mutation of the *OsDGD1* gene disrupted the lipid composition and hampered plant growth and PUE. The *osdgd1* mutation also limited Pi acquisition, probably due to the reduced root growth and biomass caused by high auxin and JA levels, which resulted from the imbalance in the MGDG to DGDG ratio (Fig. 1). These findings demonstrate the critical role of *OsDGD1* in maintaining favorable JA levels and root growth during Pi deficiency (Pandey et al. 2024).

## Data Availability

All data are incorporated into the article and its online supplementary material.
